# Molecular Aspects of Gall Formation Induced by Mites and Insects

**DOI:** 10.3390/life13061347

**Published:** 2023-06-08

**Authors:** Alexey G. Desnitskiy, Philipp E. Chetverikov, Larissa A. Ivanova, Igor V. Kuzmin, Sebahat K. Ozman-Sullivan, Sogdiana I. Sukhareva

**Affiliations:** 1 Department of Embryology, Saint-Petersburg State University, 199034 Saint-Petersburg, Russia; adesnitskiy@mail.ru; 2 Zoological Institute, Russian Academy of Sciences, 199034 Saint-Petersburg, Russia; 3 Department of Invertebrate Zoology, Saint-Petersburg State University, 199034 Saint-Petersburg, Russia; s_sukhareva@mail.ru; 4 X-BIO Institute, Tyumen State University, 625003 Tyumen, Russiai.v.kuzmin@utmn.ru (I.V.K.); 5 Department of Plant Protection, Faculty of Agriculture, Ondokuz Mayis University, 55139 Samsun, Turkey; sozman@omu.edu.tr

**Keywords:** eriophyoid mites, tenuipalpids, galling arthropods, gene expression, inducing stimulus, leaf gallogenesis, parasite–host interactions

## Abstract

Recent publications on gall formation induced on the leaves of dicotyledonous flowering plants by eriophyoid mites (Eriophyoidea) and representatives of four insect orders (Diptera, Hemiptera, Hymenoptera, Lepidoptera) are analyzed. Cellular and molecular level data on the stimuli that induce and sustain the development of both mite and insect galls, the expression of host plant genes during gallogenesis, and the effects of these galling arthropods on photosynthesis are considered. A hypothesis is proposed for the relationship between the size of galls and the volume of secretions injected by a parasite. Multistep, varying patterns of plant gene expression and accompanying histo-morphological changes in the transformed gall tissues are apparent. The main obstacle to better elucidating the nature of the induction of gallogenesis is the impossibility of collecting a sufficient amount of saliva for analysis, which is especially important in the case of microscopic eriophyoids. The use of modern omics technologies at the organismal level has revealed a spectrum of genetic mechanisms of gall formation at the molecular level but has not yet answered the questions regarding the nature of gall-inducing agents and the features of events occurring in plant cells at the very beginning of gall growth.

## 1. Introduction

Among the herbivorous arthropods, there are a considerable number of species from six orders of insects (Coleoptera, Diptera, Hemiptera, Hymenoptera, Lepidoptera, Thysanoptera), and acariform mites (Eriophyoidea: Eriophyidae, Phytoptidae, and Tetranychoidea: Tenuipalpidae), that induce the growth of galls or cecidia—specialized structures that develop on various organs of flowering plants, especially leaves. Galls develop from the tissues of the host plant and provide the phytophagous parasites inside them with nutrition and protection from predators and adverse external conditions. The estimated total number of galling insect species ranges from 13,000 to 211,000 [1,2,3]. In addition, more than 500 mite species cause gall formation [4]. The ability to induce gall formation in different phylogenetic lineages of flowering plants has arisen independently and numerous times in different phylogenetic lineages of arthropods [5,6,7,8,9]. Historically, the majority of research in this area concerns insect galls, whereas gall induction by mites has drawn much less attention. Despite the very intensive experimental study of arthropod galls [3,10,11,12,13,14], the molecular mechanisms of their formation are not fully understood yet. This can be explained in part by the fact that there is no universal model system for studying gall induction under natural conditions.

The aim of this review is to discuss the published data on the molecular aspects of gall formation in flowering plants initiated by the eriophyoid mites (Acariformes, Eriophyoidea), as well as representatives of four insect orders (Diptera, Hemiptera, Hymenoptera, and Lepidoptera). Information on the other three groups of galling arthropods listed above (Coleoptera, Thysanoptera, Tenuipalpidae) could not be included because the literature lacks molecular genetic data on gallogenesis induced by these taxa. The present paper differs from a recent review on the molecular aspects of insect galling [3] in several ways. Those authors considered galls induced by Diptera, Hemiptera, and Lepidoptera, but not those induced by Hymenoptera and eriophyoid mites, although relevant molecular data were available, e.g., [15,16]. Progress to a broader and deeper understanding of gallogenesis requires the discussion of molecular data gained from the maximum possible number of arthropod groups. The characteristics of the process of gall development vary substantially, depending on which specific arthropod is infesting the host plant [8,17,18]. Therefore, it is highly relevant to compare and contrast the development of mite and insect galls. 

In this review, after a brief survey of the functional diversity of leaf galls, the stimuli that are primarily responsible for the induction of gall development, the multistep nature of gallogenesis, links to plant gene expression, and finally the relationship between photosynthesis and leaf gallogenesis are considered in sequence. Herein, we do not discuss the symbiosis of galling arthropods and plants but focus mainly on genetic, biochemical, and histo-morphological differences between several relatively completely investigated model gall systems. The galling arthropods essentially act as “ecosystem engineers” [19]. They synchronize their life cycles with their individual host plant and in that context manipulate the host’s morphogenesis to create a niche for development and reproduction. This interesting topic deserves further investigation, preferably in combination with the recent revelations in plant molecular genetics.

## 2. Functional Diversity of Leaf Galls Induced by Arthropods

**Figure 1:**
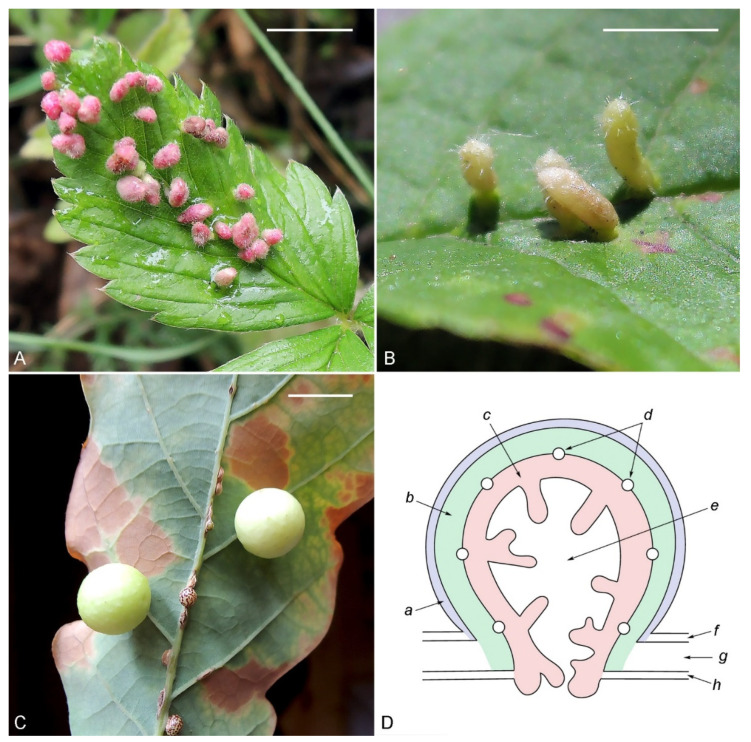
Examples of leaf galls induced by eriophyoid mites (**A**,**B**) and gall wasps (**C**), and a generalized diagram of an arthropod leaf gall with an internal chamber (**D**). (**A**)—galls of the mite, *Fragariocoptes setiger* (Nalepa, 1894), on green strawberry, *Fragaria viridis* (Weston, 1771); (**B**)—galls of the mite, *Eriophyes padi* (Nalepa, 1889), on bird cherry (*Prunus padus* L., 1753); (**C**)—galls of the oak gall wasp, *Cynips quercusfolii* (L., 1758), on petiolate oak (*Quercus robur* L., 1753). The scale bar represents 5 mm (**A**,**B**) and 10 mm (**C**). Designations: a—abaxial epidermis of the gall, b—transformed mesophyll of the gall, c—typical nutritive tissue, d—conductive bundles of the gall, e—gall chamber, f—normal abaxial leaf epidermis, g—normal leaf mesophyll, h—normal adaxial leaf epidermis.

Leaf galls on flowering plants are highly diverse in appearance, shape and color (Figure 1). Many attempts have been made to classify galls induced by both eriophyoid mites, e.g., [20,21] and insects, e.g., [22,23,24]. No general theory explains the variability of the leaf galls induced by arthropods. The classification of galls could play an important role in defining the general patterns of the molecular mechanisms of arthropod gallogenesis.

There is an important similarity in gallogenesis in the eriophyoid mites (superfamily Eriophyoidea, Acariformes), flies of the family Cecidomyiidae (Diptera), wasps of the family Cynipidae (Hymenoptera) and many butterflies (Lepidoptera). During the galling process, stimulation due to parasite feeding causes the formation of typical nutritive tissue rich in carbohydrates, proteins and/or lipids. It lines the gall chambers and serves as a direct food source for the insect and mite parasites inside [8,25]. Hesse [26,27] studied leaf galls in 60 parasite–plant pairs (mainly hymenopterocecidia, acarocecidia, and dipterocecidia) and showed the occurrence of polyploidization of the typical nutritive tissue cells in approximately half of these pairs. The degree of endopolyploidy usually increased from the periphery of the gall towards the parasite. Polyploidization occurred after synchronous mitoses without cell wall formation or after anaphase arrest that led to restitution nuclei. Much more recently, Harper et al. [28] detected the formation of polytene chromosomes in the internal cells of galls induced by the wasp *Biorhiza pallida* (L., 1758) (Cynipidae) on the leaves of the oak *Quercus robur* (L., 1753) (Fagaceae). Later, a phenomenon was discovered that is seemingly an alternative to endopolyploidy or polyteny in gall cells. The typical nutritive tissue of some galls induced on the leaves of the tree *Copaifera langsdorffii* (Desf., 1821) (Fabaceae) by a Neotropical gall midge fly (Cecidomyiidae, species not identified) contained anucleated cells [29]. Also, a Neotropical moth (Lepidoptera, species not identified) induces galls on the leaves of the woody plant *Tibouchina pulchra* (Cogn., 1885) (Melastomataceae), during whose development the cell nuclei of the nutritive tissue are lost [30]. In the typical nutritive tissue of eriophyoid mite galls, the polytene chromosomes or anucleated cells have not yet been detected. 

The functional diversity of galls induced by representatives of various families of the order Hemiptera deserves special attention. Typical nutritive tissue is not formed in galls induced by aphids (Aphididae) and jumping plant lice (Psyllidae) [8,31,32]. In such galls, the typical nutritive tissue is absent, but there is the so-called “nutritive-like tissue”, which surrounds the gall chambers. This tissue is not a source of food for the parasites inside. An attempt has been made to explain this situation as being attributable to the feeding habits of the aphids and psyllids, which do not scrape or chew plant material, as do the larvae of the gall-forming Diptera and Lepidoptera [33], but instead suck nutrients (phloem sap) directly from the conductive bundles [8,34]. However, in the galls induced by another representative of the order Hemiptera, namely the phylloxeran (Phylloxeridae) pests of grapes, which also feed on the phloem sap, the typical nutritive tissue is present [13,35,36]. 

The typical nutritive tissue, which appears in the early stages of gall formation induced by eriophyoid mites and insects, arises from the transdifferentiation (metaplasia) of leaf epidermal cells and/or the leaf parenchyma of the host plant [35,37,38]. The molecular and cellular mechanisms of this process, which can vary for different parasites, are not yet well understood. 

## 3. Stimuli That Induce the Development of Galls

The first interactions of gall-forming parasites with their host plants occur in different ways, depending on the arthropod group [8]. In case of the galling eriophyoid mites (Eriophyoidea), aphids (Aphididae) and phylloxerans (Phylloxeridae), the primary gall-inducing stimulus is produced by females when they commence feeding on young plant leaves. Since the main period of gall formation in the Palearctic region occurs on young leaves in the spring, leaf age apparently plays an important role, but this phenomenon has not been specially studied. 

The minute eriophyoid mite (body length: 100–300 μm) attacks a single epidermal cell of the host plant by piercing it with stylets and injecting saliva. The damaged cell dies, but the gall-forming effect then spreads to the adjacent leaf area, probably through plasmodesmata and the conducting system [20,37,39]. At the same time, it remains unclear whether what occurs is the dispersal of the primary, gall-inducing agents of the mite saliva, or the compounds that are synthesized by the attacked cell, or both. The leaf galls induced by eriophyoid mites are usually much smaller than the leaf galls of insects. It is reasonable to hypothesize that this is due to the diminutive size of the eriophyoids and the extremely small amount of their saliva that enters the plant cell at the earliest stages of gall formation. The tiny, needle-like stylets of mites have a much less damaging effect on plant tissues than those of the insects. During gallogenesis due to wasps, flies and butterflies, the large piercing apparatus of an adult insect or the powerful jaws of a chewing larva kill a whole group of cells at once, and the size of their salivary glands and the volume of injected saliva are an order of magnitude greater than those of eriophyoid mites. The hypothesis of a correlation between the sizes of gall initiators, the volume of agents injected by them, and the sizes of the galls formed needs experimental verification. 

In the case of gall wasps of the family Cynipidae (Hymenoptera), the primary gall-inducing signal is not the feeding of sexually mature individuals, but the laying of eggs in the leaf tissue [8]. In the process of oviposition, secretions of the venom glands of the female or her ovaries are also released, which could be the stimuli for gall formation [40]. In the case of gall-forming representatives of Diptera and Lepidoptera, the primary signal does not come from adults, but instead from larval feeding, which wounds the epidermal cells of the host [8,17,25]. In addition, not only the saliva of the larvae but also their excrement could play a role in gall initiation [3]. 

Unusual cases are known when insect larvae acquire the ability to initiate galls only after a few molts. For example, in the micromoth, *Caloptilia cecidophora* (Kumata, 1966) (Lepidoptera, Gracillariidae), which infests the leaves of the tree, *Glochidion obovatum* (von Siebold, 1845) (Phyllanthaceae), the first and second instars are leaf-miners. Their feeding produces galleries within the leaf lamina, and they do not have any gall-inducing properties. Gall induction is initiated by the third instar, which releases a cecidogenic substance that has not yet been analyzed [41]. This lepidopteran species is unable to complete its larval development of six instars without feeding on gall nutritive tissue. Interestingly, several related members of the lepidopteran genus, *Caloptilia*, are exclusively leaf-miners and they do not induce galls. Guiguet et al. [41] reasonably suggested the evolutionary transition from leaf-mining to gall-induction within this lepidopteran lineage. 

The salivary glands of a large number of species from all orders of gall-forming insects were demonstrated to contain the phytohormones, auxin and cytokinins, that control normal plant development [42,43,44,45]. Additionally, de Lillo and Monfreda [46] showed that the effects of the saliva of gall-inducing eriophyoid mites on plant tissues are similar to those produced by phytohormones. A biochemical analysis of the saliva of mites has not been done, meaning that the presence of phytohormones in their saliva has not yet been verified. The reason for this is the current impossibility of collecting a sufficient amount of the saliva of galling mites for analysis. 

The leading role in the process of gallogenesis caused by arthropods is now attributed to endogenous cytokinins [47]. On the other hand, Hearn et al. [16] reported that they did not detect the expression of genes encoding for phytohormones in the young larvae of the gall wasp, *B. pallida*. In addition, in the genome of the mite, *Fragariocoptes setiger* (Nalepa, 1894) (Phytoptidae), which causes galls on strawberry leaves, phytohormone genes and any other genes that could have entered the mite genome from the plant as a result of horizontal transfer were not found [48]. Therefore, if there are parasite–plant pairs in which gallogenesis is induced by the endogenous phytohormones of parasitic arthropods, then there are other cases, e.g., the pairs involving the wasp *B. pallida* or the mite *F. setiger*, in which gallogenesis is initiated by a different but yet unexplained mechanism. 

Since many species of eriophyoid mites and insects have symbiotic relationships with various bacteria, there is reason to suggest that along with their saliva, the host plants also receive bacteria (or their metabolic products) that cause the formation of galls [12,21,49]. In contrast, other recent studies have challenged the importance of the stimulatory effects of bacterial symbionts in the formation of galls induced by insects [16,50] and eriophyoid mites [48]. 

Important work directed towards the understanding of the molecular mechanisms involved in the initial steps in cynipid and aphid gall induction has been published recently. The transcriptome analysis of the venom glands and ovaries of the cynipid wasps *Diplolepis rosae* (L., 1758) and *B. pallida*, which induce galls on the rose, *Rosa canina* (L., 1753), and common oak, *Quercus robur*, respectively, has been performed [40]. Some maternally expressed wasp proteins (potential effectors) presumably involved in the initial parasite–host interactions were identified. They included apolipoproteins D, peroxidases, alpha-mannosidases, carbonic anhydrases and canopy 1-like proteins. There are several genes that are highly expressed in the cynipid venom gland (e.g., those encoding for acid phosphatases, apolipoprotein D, secreted peroxidases and a saposin-like protein of the Canopy 1 family) and ovaries (e.g., genes encoding for one phospholipase A2-like and two exonuclease 3′–5′ domain-like 2) that may be involved in the suppression of early plant defense signaling [40]. 

Recent molecular research on the interactions between *Hormaphis cornu* (Shimer, 1867) (Aphididae) and witch hazel *Hamamelis virginiana* (L., 1753) (Hamamelidaceae) revealed a novel aphid secretory protein that was named ‘BICYCLE’. *Bicycle* genes are strongly expressed in the aphid’s salivary glands [51]. The same group of researchers later discovered that many *bicycle* genes are strongly expressed not only in the salivary glands of a second galling aphid, *Tetraneura nigriabdominalis* (Sasaki, 1899), but also in the salivary glands of a non-gall forming aphid, *Acyrthosiphon pisum* (Harris, 1776), as well as in the non-gall forming generation of *Hormaphis cornu* [52]. Those researchers hypothesized that “these observations suggest that BICYCLEproteins may be used by multiple aphid species to manipulate plants in diverse ways” [52]: (p. 1). It is very likely that new genes and proteins involved in the initiation of arthropod gall formation will be found. Moreover, we expect that they will differ among host plant–parasite pairs. This would support the idea of independent and convergent origins and mechanisms of galling in various arthropod and plant phylogenetic lineages.

## 4. Gall Formation Is a Multistep Process

The most comprehensive experimental data on the multistep character of arthropod gall formation were obtained for the combination of the eriophyoid mite, *Eriophyes padi* (Nalepa, 1889), and bird cherry, *Prunus padus* (L., 1753) (Rosaceae) [37]. The author reported that on the control leaves, after 10 days of mite feeding, complete galls with differentiated nutritive tissue had developed. In the experiment with 8 h or 24 h contact of mites with the plant and subsequent removal of the parasites, only small primary protrusions of the leaf lamina (“abortive galls”) formed, and no further formation of galls occurred. When the mites were removed from the leaf after 48 h of contact, small pouch galls, which did not yet have typical nutritive tissue, had formed. They were therefore termed “defective galls” [37]. The dependence of the degree of gall development on the duration of the feeding period of the mite indicates a continuous transfer of gall-inducing factors produced by the mite into the plant leaf. It is possible that there is a cumulative effect, i.e., a certain critical mass of mite saliva (with gall formation inducing factors dissolved in it), injected for a certain time, is necessary for the complete development of galls. 

A multistep, cumulative effect of gall-inducing agents can also be hypothesized in the case of the development of leaf galls induced by flies of the family Cecidomyiidae [17,25]. By removing the larvae at different stages of gall morphogenesis, it was possible to analyze the succession of the host plant’s responses to parasite feeding. It was demonstrated that larval activity, which differs in nature between the first and subsequent instars, is required throughout the gall formation period. 

Thus, the primary inducing stimulus produced by the arthropod parasite, which we discussed in Section 3, is insufficient for the formation of a fully formed leaf gall, since the process must be reinforced with additional stimuli. It is not yet known whether these stimuli are simply repeats of the primary stimulus or whether they have a different nature, (e.g., the inducing effect of the parasite’s saliva being followed by the effect of its excrement or some other factor(s)). Recent molecular studies of changes in the pattern of gene expression during the development of galls induced by eriophyoid mites and hymenopteran insects indirectly support the multistep nature of leaf gallogenesis [15,16]. These two works will be considered in the next section of our review.

## 5. Expression of Plant Genes during Gall Formation

There is a reasonable body of published data on gene expression in developing gall tissues induced by the representatives of several groups of herbivorous arthropods on the leaves of various flowering, dicotyledonous plants. Table 1 provides a list of 10 host–parasite pairs studied (9 pairs involving insects and 1 pair involving an eriophyoid mite). The greatest number of articles is devoted to galls induced by representatives of the families Cynipidae and Phylloxeridae.

The eriophyoid mite *Fragariocoptes setiger* induces galls on the leaves of the green strawberry, *Fragaria viridis* (Weston, 1771) (Rosaceae). During the initiation and growth of young galls, the increase in the expression of the *CYCD3* and *CYCB1* cell cycle genes in their tissues is associated with active cell proliferation [15]. By the time of gall maturation, a sharp decrease in the expression of cell cycle genes was found. A similar dynamic of changes in gene expression during mite galling on strawberry leaves was found for the homeobox genes, *KNOX* and *WOX*. These two genes are the universal regulators of normal plant development [61]. Finally, during the development of galls on strawberry leaves, there was an abrupt change in the expression pattern of the genes responsible for the adaxial–abaxial polarity of the leaf. Specifically, a sharp increase was detected in the expression of two genes, *FviYAB2* (*YABBY2 Arabidopsis* gene homolog, abaxial side development regulator) and *FviREV* (*REVOLUTA Arabidopsis* gene homolog belonging to the HD-ZIPIII family, adaxial leaf side development regulator) [15]. At the same time, a histological analysis showed that during gall development, ventral-type tissues were formed in the dorsal part of the leaf lamina (transformed epidermis and mesophyll), a phenomenon termed “abaxialization of the leaf” by the same authors. Interestingly, a change in adaxial–abaxial polarity was also found during the development of galls induced by the grape phylloxeran, *Dactulosphaira vitifoliae* (Fitch, 1855) (Phylloxeridae) [59]. In this case, the parasite’s galling activity caused the formation of stomata on the adaxial surface of the grape leaf where stomata typically do not occur. 

The development of leaf galls induced by several wasp species of the family Cynipidae (*Amphibolips michoacaensis* Nieves-Aldrey et Maldonado, 2012, *B. pallida*, *Dryocosmus kuriphilus* Yasumatsu, 1951) when they deposit eggs in the meristematic tissues of the leaves of oaks (genus *Quercus*) and some other trees of the beech family (Fagaceae) has been studied [16,55,56,57]. These studies revealed major differences in gene expression between gall cells and the cells of normal (control) leaves, as well as changes in the expression pattern of hundreds or possibly thousands of plant genes during gall development. For example, phenylalanine ammonia lyase (PAL) enzyme genes were upregulated during the intermediate and late gall growth stages, phenylpropanoid genes were upregulated during the intermediate stage and downregulated during the late stage and lignin genes were upregulated during the late stage [55]. 

Particularly noteworthy is a study in which gene expression was analyzed during the development of both participants in a “parasite–host dialogue” involving larval *B. pallida* and the leaves of the common oak, *Q. robur*. The study demonstrated that “gall development involves expression of oak and gall wasp genes in repeatable, growth stage-specific patterns” [16]: (p. 19). In particular, in the tissues of young galls, the enhanced expression of *ENOD* genes occurred. These genes had been discovered in nitrogen-fixing nodules of a legume family (Fabaceae) and were later found in many other plants [62]. Nodulin-like proteins encoded by these genes belong to the large family of arabinogalactan proteins, which are glycoproteins involved in plant growth and development processes, including somatic embryogenesis [63,64,65]. These proteins are understood to be similar to proteoglycans, which are important for morphogenetic processes in multicellular animals and are involved in the transmission of interstitial signals [66]. 

In the young larvae of *B. pallida*, the expression of *PCWDE* genes, which code for plant cell wall degrading enzymes, including six pectin/pectate lyases, four cellulases and four rhamnogalacturonan lyases, occurs [16]. Enzymes encoded by these genes disrupt the wall structure of plant cells in this wasp’s feeding area. Then, numerous secreted peptides, including wasp chitinases, move into the gall tissues surrounding the larva, although it is not yet clear which larval tissue produces chitinases. The same authors inferred that their data supported a hypothesis, although it is not yet generally accepted, that galls induced by wasps of the family Cynipidae can be considered “modified somatic embryos”, with their development being similar to the somatic embryogenesis of plants. They stated that “host arabinogalactan proteins and gall wasp chitinases interact in young galls to generate a somatic embryogenesis-like process in oak tissues surrounding the gall wasp larvae” [16] (p. 1). During somatic embryogenesis, a fully developed fertile plant organism develops from a single somatic cell [67,68]; the molecular aspects of this process have been intensively studied [69,70,71]. In addition, somatic embryogenesis in higher plants is linked to the presence of totipotent and pluripotent cells that can dedifferentiate and transdifferentiate. These features are also necessary for the process of galling. 

A very recent study [53,54] revealed for the first time the tissue-specific gene expression in the active growth phase of young galls induced by the wasp *Dryocosmus quercuspalustris* (Osten-Sacken, 1861) on the leaves of the red oak, *Quercus rubra* (L., 1753). The analysis was carried out during a single stage of gall formation, specifically a young actively growing gall with a feeding larva in the internal gall chamber (approximately 5–6 days after oviposition). For the first time, not only were significant differences (28%) revealed between the transcriptomes of the whole gall and the adjacent leaf tissue, but also between the outer gall tissue, which performs a predominantly protective function, and the internal tissue of the gall, on which the parasite feeds. In general, the transcriptome of the outer tissue of the gall was more similar to the transcriptomes of the tissues of leaf buds, twigs and reproductive structures of oak than to the transcriptome of normal leaf tissue. In this study, as well as in the work on galls initiated by *B. pallida* [16], the active expression of the *ENOD* genes was demonstrated in both the inner and outer tissues of young galls. 

Leaf galling is also induced by some hemipterans. In the case of a parasite–plant host pair, the aphid, *Schlechtendalia chinensis* (Bell, 1851) (Aphididae), and sumac, *Rhus javanica* (L., 1753) (Anacardiaceae), in the early stages of gall formation, increased expression of the *KNOX* genes occurs [58]. In another parasite–host pair, the grape phylloxeran, *Dactulosphaira vitifoliae*, and coastal grape, *Vitis riparia* (Michaux, 1803) (Vitaceae), the genes associated with the development of reproductive structures, flowers and fruits, including *LFY*, *AG*, *SEP*, *SHP*, *CAL* and *FUL*, were activated [60]. 

An attempt to identify the genes involved in leaf gallogenesis involving the simultaneous use of several parasite–host pairs was recently undertaken by Takeda et al. [3,72]. Transcriptomes from galls formed by three pairs were studied: *Rhopalomyia yomogicola* (Matsumura, 1931) (Diptera, Cecidomyiidae)—*Artemisia montana* (Pampanini, 1930) (Asteraceae), *Caloptilia cecidophora* (Lepidoptera, Gracillariidae)—*Glochidion obovatum* (Phyllanthaceae) and *Borboryctis euryae* (Kumata et Kuroko, 1988) (Lepidoptera, Gracillariidae)—*Eurya japonica* (Thunberg, 1783) (Pentaphylacaceae). Their molecular data were discussed in combination with the molecular data obtained by the same authors for the pair *Schlechtendalia chinensis*—*Rhus javanica* [58], which we briefly reviewed above. A comparison of four transcriptomes of gall tissues showed that among the several thousand genes analyzed, there were about 40 “common genes” active in all four cases of gall development [3,72]. These included some genes encoding key peptide regulators in plant growth and development, e.g., *CLE44*, *BAM3* and *WOX* [73]. In all four variants of gall formation studied by the Takeda group [3,72], the suppression of genes associated with photosynthesis was detected. Galls induced by the moth *B. euryae* on *E. japonica* showed no activation of genes (e.g., *AG*, *SEP*, *SHP*) associated with the development of reproductive structures, whereas the galls formed by the other three parasite–host pairs showed the activation of genes involved in floral organ development. Takeda et al. [72] stated that in each of the four cases of gall development that they studied, in addition to the common set of genes for all pairs, different supplementary sets of genes were also mobilized, resulting in the creation of a unique gall structure by each pair. 

Overall, there is relatively little information on the analysis of gene expression changes during leaf galling induced by arthropods. Nevertheless, due to gene expression, galls cease to be typical leaf structures and acquire similarities to other plant organs, but each time in a different way, depending on the gall-forming plant–arthropod combination. Thus, each gall seems to be a unique structural mosaic that includes morphological elements characteristic of the normal development of vegetative and generative organs.

## 6. Gallogenesis and Photosynthesis

In the previous section, it was noted that, during gall development induced by representatives of the orders Diptera, Hemiptera, Hymenoptera and Lepidoptera, leaf gall tissues of host plants usually show a significant downregulation of the genes associated with photosynthesis [58,59,72]. The latest study compared internal and external tissues of the young galls induced by the wasp *Dryocosmus quercuspalustris* on the leaves of the oak, *Quercus rubra* [53]. The internal tissue, which was termed heterotrophic, was characterized by an increased expression of genes encoding for the synthesis of sucrose, and a complete suppression of the genes associated with photosynthesis. In addition, the outer gall tissue was green, but the expression of genes associated with photosynthesis was significantly reduced in comparison to the adjacent leaf tissue. 

Leaf galls induced by the eriophyoid mites have not yet been studied with respect to the expression of their photosynthesis-related genes. Nevertheless, in the case of mite gallogenesis, there are morphological and physiological data that indicate significant destruction of the photosynthetic apparatus (reduction in leaf area, a decrease in the chlorophyll and carotenoid content per unit of leaf area and per whole leaf), and inhibition of the process of photosynthesis [74,75]. At the same time, the formation of multiple mite galls on a leaf does not affect the concentration of chlorophyll or, apparently, the intensity of photosynthesis in the areas of the same leaf between galls that are unaffected by galling [76]. The photosynthetic activity of a leaf depends on both the leaf area and the photosynthesis rate per unit of leaf area. In several mite–host tree systems, the variation of unaffected leaf area depends on the particular mite–tree system in a severity-independent manner, whereas chlorophyll and carotenoid content in infested leaves directly correlates with the infection severity [76]. This represents the evidence of a complex mechanism of gall influence on photosynthesis in infested leaves. 

In general, gall-forming insects and eriophyoid mites have a similar suppressive effect on photosynthesis in the leaf galls induced by them on plants. A notable exception is represented by two unusual galling insect–host plant pairs consisting of the gall-forming beetle weevils, *Smicronyx smreczynskii* (Solari, 1952) or *Smicronyx madaranus* (Kôno, 1930) (Coleoptera, Curculionidae), and field dodder, *Cuscuta campestris* (Yunck., 1932) (Convolvulaceae). Field dodder is an obligate parasitic plant with a very low chlorophyll content and very weak photosynthetic activity. The weevil larvae significantly enhanced the photosynthetic activity in the spherical galls induced by them on the dodder shoots and they therefore acquired nutrient-rich shelters [77,78]. This example once again testifies to the potential diversity of the mechanisms involved in galling.

The molecular mechanisms involved in the suppression or enhancement of photosynthesis by gall-forming insects and eriophyoid mites currently remain largely unknown. These phytoparasitic arthropods inhabit the leaves of plants and it is difficult to maintain such parasite–tree systems in the laboratory to study the mechanisms involved in the suppression or enhancement of photosynthesis in gall tissues [78]. Among the reasons for the frequent decrease in the photosynthetic capacity of galled leaves may be a decline in stomatal conductance and in photosystem II efficiency [79,80,81]. The enhancement of photosynthetic activity in the leaves infested by the gall-forming beetle weevils could be a consequence of an increase in chloroplast numbers and chlorophyll content in the inner layer of galls [78].

## 7. Concluding Remarks

The gall-inducing ability of representatives of different phylogenetic lineages of arthropods in representatives of different phylogenetic lineages of plants has arisen repeatedly and independently. The primary inducing stimulus for leaf gallogenesis in most cases is oviposition by galling wasps (Hymenoptera), the larval feeding of flies (Diptera) and micromoths (Lepidoptera), and the feeding of the adult females of Hemiptera (Aphididae, Phylloxeridae, Psyllidae), eriophyoid mites (Eriophyoidea) and tenuipalpids (Tetranychoidea: Tenuipalpidae). The molecular mechanisms underlying these processes are still unclear. The latest advances in this field involved the detection of the active expression of several secreted proteins (potential effectors) in the salivary glands of gall-forming aphids [51,52], as well as in the ovaries and venom glands of galling wasps [40]. 

Changes in the pattern of expression of many genes in gall cells in the course of their development, as well as differences in gene expression between the tissues of galls and normal leaves, have been demonstrated in all the parasite–host systems studied. The use of modern omics technologies at the organism level suggests the involvement of numerous molecular genetic mechanisms in gall formation, but so far has not answered the question of the nature of gall-inducing agents and the features of events occurring in plant cells at the very beginning of gall growth. Breakthroughs in this area can be expected from the use of single cell (SC) technologies. Comparative SC proteomics, transcriptomics and metabolomics could reveal the elements of the functioning of the individual cells in the tissues of gall-forming arthropods (for example, in the gland cells of mites and insects, presumably those secreting gall-inducing factors), as well as in plant cells directly exposed to these factors. Such studies would contribute to the revelation of the entire sequence of stages of gall development. 

A general feature of arthropod gall development on plant leaves is the suppression of photosynthesis. It has also been shown that during leaf galling induced by the representatives of several orders of insects, genes associated with the development of reproductive structures are activated. Such data are not yet available for mite gallogenesis. 

The formation of arthropod galls appears to be a multistep process, all stages of which are still poorly understood. It appears that there is no universal scheme of gallogenesis, and in some cases, some steps may be skipped, e.g., the absence of the formation of typical nutritive tissue in galls induced by insects of the hemipteran families, Aphididae and Psyllidae. In the future, it will be important to conduct molecular analyses of both the initial and advanced stages of gall development induced by representatives of various groups of arthropods, including eriophyoid mites, paying special attention to the very early stages of gall growth, as well as to the mechanisms of nutritive tissue differentiation. This research would potentially yield huge economic benefits because many widely grown tree crops, such as lychees and walnuts, are currently affected by galling.

In conclusion, it should be noted that recently generated data indicate a high diversity of gall formation modes across various groups of gall-forming insects. At the same time, the data on insects are incomparably more complete than for mites; in particular, data on the molecular aspects of galling are available only for one mite–plant system [15,48]. The question therefore arises as to what data on the gall-forming insects would be most useful when planning further work on galls induced by eriophyoid mites. There is a reason to expect that mite galling, as a result of convergent evolution, is more similar to the galling caused by phylloxerans than to that caused by insects from other families. In both cases, the primary gall-inducing stimulus is produced by the miniature adult females that feed on young leaves. In addition, the phylloxeran galls, as with eriophyoid mite galls, have the typical nutritive tissue, and in the process of their formation, a change in adaxial–abaxial polarity occurs [15,59]. 

Eriophyoid mites have as long a history of association with plants as insects. This superfamily is an ancient lineage of chelicerates related to worm-like, soil nematalycide mites, which adopted phytophagy even before the appearance of gymnosperms and flowering plants. Some gall-like structures on the fossils of extinct plants, attributed to the activity of these mites, date back to the Late Paleozoic [48,82,83,84,85,86]. Recent representatives of many phylogenetic lineages of eriophyoids are capable of inducing galls of diverse structure on phylogenetically unrelated plants in all climatic zones of the planet. Nevertheless, histomorphological data, and even more so, molecular data, are lacking for the majority of eriophyoid galls. The same, but to an even greater extent, is true of tetranychoid mites of the family Tenuipalpidae, the second group of mites capable of gall induction. Species from seven genera (*Brevipalpus*, *Capedulia*, *Dolichotetranychus*, *Larvacarus*, *Obdulia*, *Obuloides*, *Phytoptipalpus*) out of about 40 tenuipalpid genera live in bark and leaf galls [4,87,88,89,90,91]. It is likely that with an increase in the number of thoroughly studied gall systems involving mites, a high diversity of types of gall formation will be discovered. Additional groups of mites and insects that have converged in the mechanisms and the morphological features of their gall formation will almost certainly be identified. The question of the causes of such convergence will likely attract increased research interest.

## Figures and Tables

**Table 1:** List of selected gall-forming arthropod–plant pairs in which gene expression in developing gall tissues has been studied over the past 10 years.

Taxonomic Position of Parasitic Species	Arthropod Species	Host Plant Species	References
Acariformes, Eriophyoidea, Phytoptidae	*Fragariocoptes setiger*	*Fragaria viridis* (Rosaceae)	[15]
Insecta, Hymenoptera, Cynipidae	*Biorhiza pallida*	*Quercus robur* (Fagaceae)	[16]
	*Dryocosmus quercuspalustris*	*Quercus rubra* (Fagaceae)	[53,54]
	*Amphibolips michoacaensis*	*Quercus castanea* (Fagaceae)	[55,56]
	*Dryocosmus kuriphilus*	*Castanea mollissima* (Fagaceae)	[57]
Insecta, Hemiptera, Aphididae	*Schlechtendalia chinensis*	*Rhus javanica* (Anacardiaceae)	[58]
Insecta, Hemiptera, Phylloxeridae	*Dactulosphaira vitifoliae*	*Vitis riparia* (Vitaceae)	[59,60]
Insecta, Diptera, Cecidomyiidae	*Rhopalomyia yomogicola*	*Artemisia montana* (Asteraceae)	[3]
Insecta, Lepidoptera, Gracillariidae	*Caloptilia cecidophora*	*Glochidion obovatum* (Phyllanthaceae)	[3]
	*Borboryctis euryae*	*Eurya japonica* (Pentaphylacaceae)	[3]

## Data Availability

Not applicable.
